# Mechanical and biological properties of 3D printed bone tissue engineering scaffolds

**DOI:** 10.3389/fbioe.2025.1545693

**Published:** 2025-04-04

**Authors:** Mingxuan Wang, Yunpeng Xu, Luoxi Cao, Le Xiong, Depeng Shang, Yang Cong, Dan Zhao, Xiaowei Wei, Junlei Li, Dapeng Fu, Haoyi Lian, Zhenhua Zhao

**Affiliations:** ^1^ Orthopaedic Department, Affiliated ZhongShan Hospital of Dalian University, Dalian, Liaoning, China; ^2^ Orthopaedic Medical Research Center, Dalian University, Dalian, Liaoning, China

**Keywords:** 3D printing, biological scaffolds, mesenchymal stem cell, bone defect, bone tissue engineering, biocompatibility, mechanical strength 3D printing, mechanical strength

## Abstract

Bone defects have historically represented a significant challenge in clinical practice, with traditional surgical intervention remaining the gold standard for their management. However, due to the problem of the origin of autologous and allogeneic bone and the complex and diverse bone defects, traditional surgical methods sometimes cannot meet the treatment needs and expectations of patients. The development of bone tissue engineering and 3D printing technology provides new ideas for bone defect repair. Ideal bioscaffold materials must have good mechanical properties, biocompatibility, osteoinduction and bone conduction capabilities. Additionally, factors such as degradation rate, appropriate porosity and a sustained antibacterial effect must be taken into account. The combination of 3D printing technology and synthetic composite biomaterial scaffolds has become a well-established approach in the treatment of complex bone defects, offering innovative solutions for bone defect repair. The combined application of seed cells, signalling factors and biological scaffolds is also beneficial to improve the therapeutic effect of complex bone defects. This article will therefore examine some of the most commonly used 3D printing technologies for biological scaffolds and the most prevalent bioscaffold materials suitable for 3D printing. An analysis will be conducted on the mechanical and biological properties of these materials to elucidate their respective advantages and limitations.

## 1 Introduction

Trauma, inflammation, tumour and osteoporosis are the main causes of bone defects. At present, there are many surgical methods for the treatment of bone defects, and the traditional surgical methods mainly include autologous or allogeneic bone transplantation, Masquelet technique and Ilizarov technique (Zhu et al., 2022). Autologous bone grafting is considered the gold standard for the treatment of bone defects (Myeroff and Archdeacon, 2011), which has the characteristics of less immune rejection and good histocompatibility. However, the source of autologous bone is limited, and there is a risk of complications such as pain, inflammation, and nonunion at the bone donor site. The Masquelet membrane induction technique has been shown to be effective in the treatment of long bone defects, but the treatment is long and requires two operations (Zhu et al., 2022). The Ilizarov femoral transport technique requires few bone and skin grafts and has a wide range of applications, but it takes a long time for the bone to heal and complications can occur during the healing process (Jin and Aihemaitijiang, 2021). The treatment of critical size bone defects with irregular shapes remains a major challenge in the field of orthopedics (Zhou et al., 2024). Bone tissue engineering was first proposed by Crane et al.,which mainly includes seed cells, biological scaffolds and signaling factors. It provides a new idea for the treatment of bone defects.3D printing technology emerged in the 1980s,and 3D printed biological scaffolds have the advantages of fast molding speed and high precision, which can be applied to complex bone defects (Mirkhalaf et al., 2023). 3D printing technology is reshaping the paradigm of bone regeneration through material innovation, bioactive integration and clinical transformation. It’s core value lies not only in solving the limitations of traditional treatment, but also in promoting regenerative medicine from “substitute repair” to “functional reconstruction”, and at the same time giving rise to the upgrading of the industrial chain and multidisciplinary cross-innovation (Ganapathy et al., 2022; Wu et al., 2023). In the future, with further breakthroughs in intelligent printing and environmentally friendly materials, 3D printing is expected to realize wider clinical application and promotion in the field of bone regeneration. However, the current common scaffold materials often cannot meet all the requirements in terms of mechanical and biological properties (Fallah et al., 2022), and the ideal biological scaffold materials need to be explored.

## 2 3D printing technology

**FIGURE 1:**
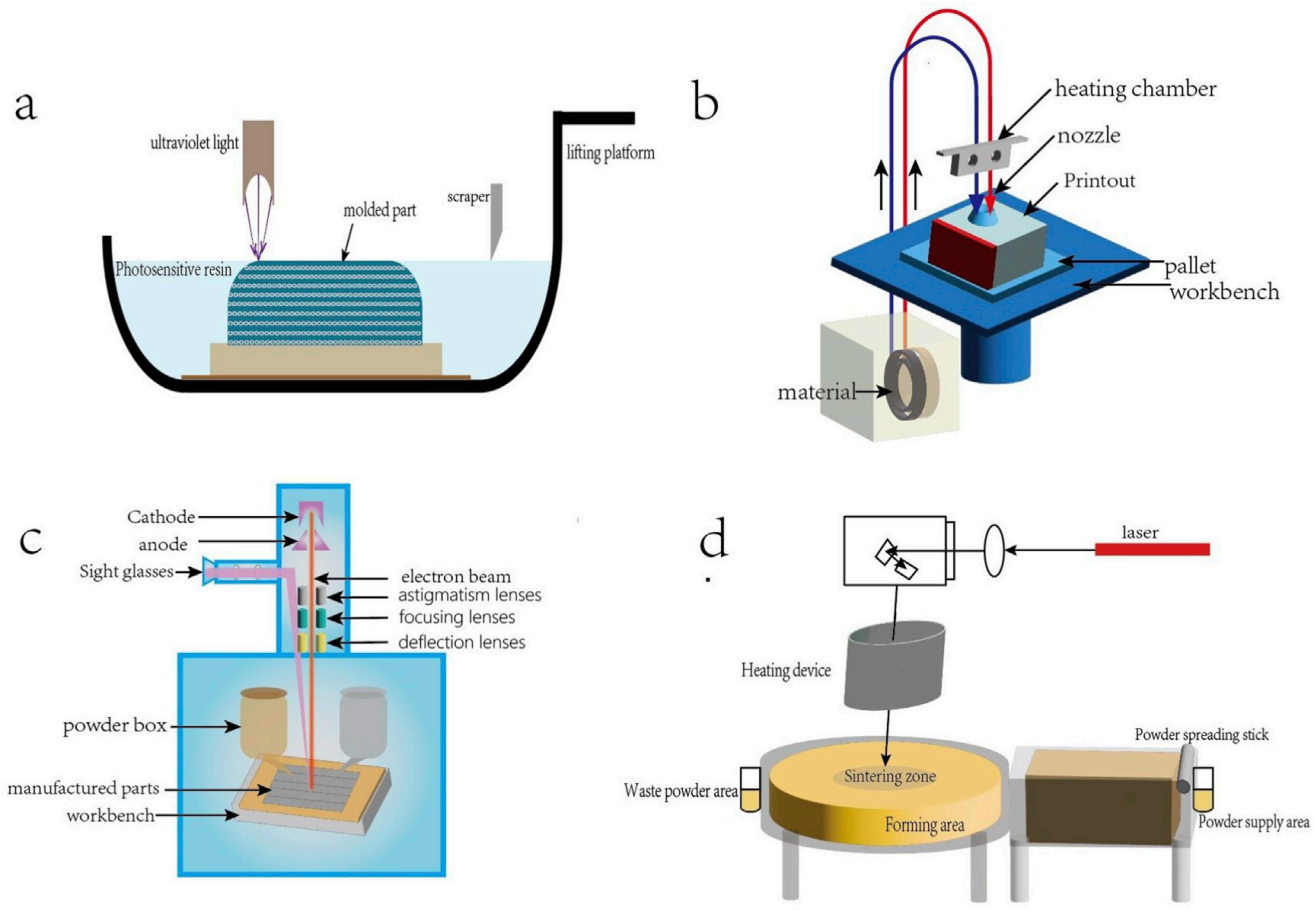
**(a)** Light-curing 3D printing technology, the use of ultraviolet laser or a specific wavelength light source selective irradiation of the surface of the liquid photosensitive resin, so that it is cured layer by layer molding. **(b)** FDM process:filaments are fed into the heated nozzle, melted and extruded; the nozzle moves in accordance with the preset path, the deposition of molten material to form a single layer, the platform descends to build up layer by layer to completion. **(c)** EBM process: the powder spreading roller spreads metal powder evenly on the platform, the electron beam scans according to the preset path, melts the powder to form a dense layer, the platform descends, and repeats the powder spreading and melting until completion. **(d)** SLS process: the powder spreading roller spreads the powder on the platform, the laser scans the powder surface, the sintered particles form a monolayer, the platform descends, and the powder spreading and sintering are repeated until completion.

3D printing technology, also known as rapid prototyping technology, is a technology that uses computer-aided design to construct objects by printing materials layer by layer. 3D printing was first proposed by Charles Hull in the 1980s, and new technologies based on 3D printing have been continuously developed for application in living organisms (Qin et al., 2024). In the field of bone tissue engineering, 3D printing offers the advantages of rapid speed, high precision and personalized customization (Liu et al., 2024), which enables the production of biological scaffolds that can accommodate the diverse requirements of bone defects in terms of shape and length. After decades of development, 3D printing technology has been continuously developed and put into practice, mainly including laser-assisted printing, fused deposition modeling (FDM), selective laser sintering (SLS), electron beam melting (EBM) and other methods. Depending on the difference in the physical state of the printing material, the appropriate method can be selected. Figure 1 provides a schematic representation of the various 3D printing technologies.

### 2.1 SLS technology

SLS is a processing technology based on powder materials, which uses the energy of the laser beam to heat and fuse powder particles to form a solid structure (Sun, 2020). The parameters of SLS are dependent on the physical and chemical properties of the materials used, and in some instances, the molded parts cannot be formed directly by SLS and require reprocessing (Zeng et al., 2018). Due to the existence of a certain amount of voids between the powders, this will affect the microstructure and physicochemical properties of the printed scaffold, resulting in the decrease of its density and mechanical properties (Deng et al., 2023). SLS technology does not require supporting structures and can be printed in batches. However, powder processing is time-consuming and mainly relies on industrial grade equipment, resulting in slightly higher costs. SLS technology can be used for the fabrication of bioceramic and thermoplastic material scaffolds. The hydroxyapatite (HA)/polylactic acid (PLA) scaffolds fabricated by this technology have good shape plasticity, suitable pore structure, ideal mechanical properties and cellular compatibility (Zeng et al., 2018).

### 2.2 FDM technology

FDM is a method of constructing a 3D structure by depositing thermoplastic materials on a substrate in layers using a temperature-controlled print head (Winarso et al., 2022). The FDM process does not require a laser, which has the advantages of low cost, simple molding equipment and small size (Ni, 2013), and can significantly shorten the manufacturing cycle of printed molded parts (Yuan, 2023). However, this technique has certain limitations due to the limited variety of biocompatible materials available and the difficulty in establishing printing parameters when printing bone scaffolds with high mechanical properties (Winarso et al., 2022). The primary materials commonly employed in FDM technology are PLA and acrylonitrile butadiene styrene, with an increasing number of other materials being utilised for the fabrication of biological scaffolds (Melcova et al., 2020). The printing speed of melt deposition molding technology is moderate, mainly limited by the nozzle movement speed and layer thickness. Its equipment has low cost, simple operation, and is widely used in education and small and medium-sized enterprises. There are unique advantages in the preparation of low-cost medical devices such as orthotics and external fixation brackets.

### 2.3 EBM technology

EBM is a method of using a high-energy electron beam to melt metal powder, and through melting layers, it is deposited to create the required part (Wang et al., 2013). The process needs to be carried out under vacuum conditions, which not only ensures the high purity of the EBM to the finished part, but also reduces the risk of hydrogen absorption. In addition, the temperature of about 700°C is maintained to reduce the deformation and warping of the part (Zheng, 2017). The advantages of EBM technology include material savings, low cost, a simple procedure and high accuracy (Mao et al., 2016). It has significant advantages in the preparation of titanium alloy porous bone scaffolds, and can accurately match the shape of defects. However, it should also be noted that the equipment is expensive, requires a vacuum environment, and the production cost of the bracket is high. Currently, it is only used in high-end industrial and medical fields.

### 2.4 Light-curing 3D printing technology

Light-curing 3D printing technology uses computer-controlled laser beams or digital light to selectively cure photosensitive materials, stacking them layer by layer to form customized 3D structures (Jiang et al., 2023), common types include stereolithography appearance, digital light processing and two-photon polymerization lithography (Zhou et al., 2023). The advantages of light-curing 3D printing include good surface quality, high utilisation of raw materials, low energy consumption, a short production cycle, a wide production area and high printing accuracy (Wang et al., 2024). Furthermore, it can quickly print complex structures with high resolution (Rajput et al., 2022). Light-curable printing technology can be used for 3D printing of a variety of materials, including materials such as hydrogels, bioceramics, and some metal powders. At present, photolithography is applicable to a range of tissues, including blood vessels and cartilage. Light-curing 3D printing equipment is widely available and reasonably priced, offering high precision but relatively longer layer curing times, making it suitable for small-batch, intricate models. In clinical applications, it is commonly used to fabricate surgical guides and customized models, enabling high-precision printing of patient-specific anatomical structures to assist in preoperative planning.

### 2.5 Bioink and biomaterial ink

Bioink is a key material used for biological 3D printing, mainly for constructing *in vitro* models of cells, tissues or organs. It possesses the characteristics of biocompatible, printable and support cell growth (Habib and Khoda, 2022). According to Groll et al.'s proposal, bioink is defined as a cellular formulation suitable for processing by automated biomanufacturing techniques, which may also contain bioactive components and biomaterials (Groll et al., 2018). Meanwhile, Groll et al. refer to biomaterials that can be inoculated with cells directly after printing, rather than formulated directly with cells, as biomaterial inks. There are a wide range of types of biomaterials ink,including thermoplastic polymers such as polycaprolactone; non-biodegradable polypropylene; biopolymers such as gelatin; and inorganic materials such as adhesives and metals. Both materials have important applications in tissue engineering and regenerative medicine. Such as repairing or replacing defective tissues, cartilage and blood vessels, and printing functional organ prototypes in combination with stem cell technology. Although their applications currently face challenges, shear forces during the printing process may damage cells, complex tissues require vascular networks to support nutrient delivery, and material batch differences affect clinical applications. But it has shown great potential in tissue engineering and regenerative medicine (Lin et al., 2022). There is significant potential in several areas, such as smart materials like dynamic hydrogels that respond to environmental stimuli, sustainable development through the creation of biodegradable inks to reduce environmental impact, and industrial integration by combining artificial intelligence to optimize printing parameters and advance personalized medicine.

## 3 Mechanical and biological properties of 3D-printed biological scaffolds

### 3.1 Metal materials

Common metal scaffold materials include titanium, copper, tantalum(Ta), silver, magnesium, zinc(Zn), iron and so on. The advantages of metal scaffold materials are good corrosion resistance, suitable mechanical properties and high mechanical strength. Some metal scaffolds are gradually degraded *in vivo*, and the released metal ions can affect mesenchymal stem cells (MSCs), osteoblasts and endothelial cells(ECs), which is conducive to their osteogenic differentiation and the formation of new capillaries. In addition, some metal ions have excellent antibacterial properties and play an important role in promoting bone tissue growth. SLS and EBM techniques are suitable for the preparation of metal supports. However, we also need to pay attention to the problems of poor biocompatibility and cytotoxicity of metal scaffold materials. Table 1 summarizes the mechanical properties of common metal materials.

**TABLE 1:** Mechanical properties of metal materials.

Material	Density (g/cm^3^)	Degradability	Modulus of elasticity (GPa)	Yield strength (MPa)	Tensile strength (MPa)
Human Bone	1.87 ∼ 1.97	——	Cortical bone: 17 ∼ 20 Cancellous bone: 3.2 ∼ 7.8	Cortical bone: 100 ∼ 190 Cancellous bone: 1.5 ∼ 10	Cortical bone: 130 Cancellous bone: 2 ∼ 12
Titanium	4.506	Non-biodegradable	105∼109	140	235
Tantalum	16.65	Non-biodegradable	186∼191	120∼300	309
Magnesia	1.74	Degradability	41∼45	65 ∼ 100	165∼205
Zinc	7.14	Degradability	130	90 ∼ 100	110∼150

#### 3.1.1 Titanium

**FIGURE 2:**
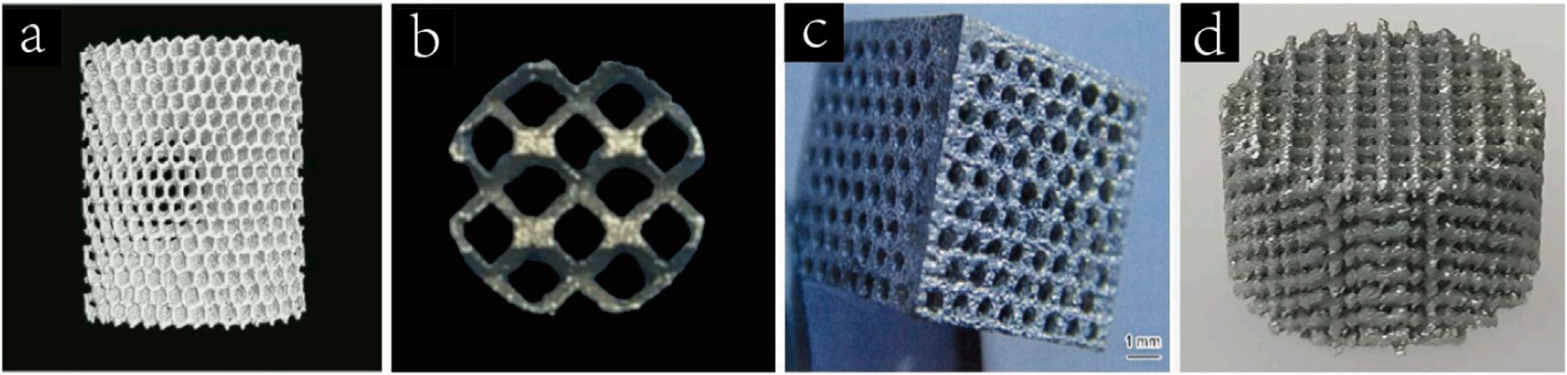
**(**a**)** Porous titanium scaffolds fabricated by SLS (Wang et al., 2023a). Copyright 2017, Elsevier. **(b)** Porous tantalum(pTa) scaffold treated by SLS (Zhao et al., 2021). Copyright 2021, Oxford University Press. **(c)** Porous magnesium scaffold prepared by laser drilling technology(Yazdimamaghani et al., 2017). Copyright 2017, Elsevier. **(d)** 3D printed porous Zn scaffold (Xia et al., 2023). Copyright 2020, Elsevier.

Titanium is the most commonly used metal scaffold for bone defect repair, with Ti6Al4V being the predominant alloy. Figure 2a shows the preparation of porous titanium scaffold by SLS. Compared with other metal scaffolds, titanium alloy has the advantages of low density, strong corrosion resistance and low biological toxicity, which makes it play an important role in the application of bone defects. Titanium scaffolds are usually prepared in the form of porous titanium alloy. The porosity of the material is more conducive to the early adhesion and proliferation of cells and is conducive to the repair of bone defects. Many studies have shown that porous titanium scaffolds with porosity of 60%–70% have similar mechanical strength to human trabecular bone (Taniguchi et al., 2016). The bioinert nature of titanium alloys limits their ability to achieve rapid osseointegration. However, appropriate porosity and surface morphology can improve osseointegration while providing a pathway for cell ingrowth and material transport (Wang, 2023; Yang et al., 2023).

In addition to the excellent mechanical properties, the biological properties of titanium alloy materials have also been widely concerned. Zhang et al. demonstrated porous titanium’s bone-forming capacity equals hydroxyapatite scaffolds (Zhang et al., 2011). Phuoc et al. also transplanted the prepared Ti6Al4V porous implant into a model of tibial diaphyseal bone defect in New Zealand white rabbits (Phuoc et al., 2023). The results also showed that cortical bone could grow inwards onto porous Ti6Al4V, and the porous titanium alloy material had good osseointegration ability. In addition to porous structures, bone formation can also be promoted by photostimulation, ultrasound induction, and pH response (Zhang et al., 2024). Titanium implants lack antimicrobial activity (Li, 2019). In recent years, many methods have been used to improve the antibacterial performance of titanium alloy scaffolds. Yu et al.effectively eliminated the biofilm formed by methicillin-resistant *Staphylococcus aureus* on titanium implants by combining photothermal and NO treatment, which can effectively reduce the risk of infection (Yu et al., 2023). Wang et al. loaded vancomycin into the hydrogel and implanted it into the titanium stent, which significantly improved the antibacterial ability of the scaffold (Wang, 2023). The microstructure of the 3D-printed titanium scaffold surface promoted the proliferation, differentiation, and mineralization of MC3T3-E1 pre-osteoblasts at an early stage compared with the polished surface (St-Pierre et al., 2005). Bassous et al. also found that scaffolds with rough and wet surfaces have better ability to promote osteoblast adhesion and proliferation (Bassous et al., 2019).

Porous titanium has been extensively studied as a bone substitute material for a long time, is widely applied, offers moderate pricing, and has been clinically validated through practical use. Due to its excellent mechanical properties, safety and biocompatibility, it has a good application prospect. However, porous titanium materials have weak osteogenic ability, non-degradability and poor antibacterial ability, which also limit their application.

#### 3.1.2 Tantalum

Similar to titanium, Ta is an inert metal that forms a dense oxide layer on its surface when exposed to oxygen, exhibiting near-insolubility in acidic environments. Figure 2b shows a pTa scaffold treated by SLS. pTa scaffolds are characterized by high porosity, low modulus of elasticity, high fatigue strength and appropriate mechanical properties, which ensure their long-term stability after implantation (Peng et al., 2022). Jiao et al. prepared tantalum scaffolds with 60%, 70%, and 80% porosity using laser powder bed fusion and implanted them into rat femoral defect models. pTa scaffolds with 70% porosity demonstrated optimal osteogenesis, osteoconduction, osseointegration, biosafety, and mechanical performance (Jiao et al., 2023).

In addition to the suitable mechanical properties, the excellent biological properties of pTa are also the reasons why it can be used as a biological scaffold. There are two mechanisms by which tantalum promotes osteogenic differentiation: (1) pTa has good cell adhesion; (2) regulation of related genes and activation of signalling pathways (Zhou and Liu, 2022). The 3D structure and good biocompatibility of pTa materials are beneficial to the adhesion and proliferation of BMSCs and osteoblasts (Geng et al., 2014). pTA scaffolds can promote osteogenic differentiation of BMSCs, which may be due to their activation of the MAPK/ERK signaling pathway, thereby regulating the expression of osteogenic genes OSX, Col-I, OSN and OCN (Dou et al., 2019). Some studies have found that the addition of a tantalum coating on the surface of titanium tubes accelerates the rate of matrix mineralization and bone nodule formation by 30% (Frandsen et al., 2014). pTa scaffolds can also promote the formation of new capillaries. When BMSCs-derived ESc were cultured on the surface of a pTa-GNPS hydrogel scaffold, the formation of capillary-like network was significantly accelerated, indicating the angiogenic properties of the scaffold (Zhao et al., 2021). Wei et al. designed a model of left hind limb osteochondral defect in male goats. It was found that both the pTa scaffold alone and the COL membrane/pTa composite scaffold promoted the generation of new bone tissue (Wei et al., 2020). In addition, porous tantalum may also exert osteogenic induction through signaling pathways such as integrin/FAK/ERK1/2, Wnt/β-catenin, TGF-β/Smad, and autophagy pathway (Zhou and Liu, 2022).

Tantalum is a promising biological scaffold material due to biocompatibility, mechanical properties, osteogenic differentiation of MSCs and osteoblasts, formation of new capillaries and good corrosion resistance. However, it should also be noted that tantalum is expensive, ranging from $300 to $600 per kilogram, and is non degradable with poor antibacterial properties (Xu, 2023).

#### 3.1.3 Magnesia(Mg)

Unlike titanium and tantalum, Mg is a more reactive metal that can gradually degrade in the body (Yan et al., 2022). The density of magnesium is 1.74∼2.0 g/cm^3^,which is closer to the density of human dense bone tissue(1.87∼1.97 g/cm^3^) than other metal implant materials. Its modulus of elasticity is 41∼45 GPa, and its compressive yield strength is 65–100 MPa. Figure 2c shows porous magnesium scaffolds prepared by laser drilling technology. Mg^2+^ is an important metal element in the human body, with an average adult containing about 24 g of magnesium, of which 53% of Mg^2+^ is stored in bones (Zhang et al., 2021).

Mg degrades in the body to promote the deposition of calcium and phosphorus, which is then converted into bone tissue. At the same time, Mg can also act as a cofactor to promote bone formation. After the degradation of Mg alloys, the local high Mg^2+^ environment can promote osteoblast adhesion, proliferation and mineralization and inhibit the bioactivity of osteoclasts (Ma, 2017). However, excessive Mg^2+^ concentration inhibited the proliferation of osteoblasts, and ALP activity and OCN expression were upregulated and the osteoblast proliferation rate increased in the environment of 0.5mM∼4 mM Mg^2+^, while the opposite was observed at 8mM and 16 mM (Lu et al., 2017). Mg^2+^ concentration also affects ECs proliferation, with high concentrations of Mg^2+^ stimulating the synthesis of angiogenic factors, attenuating lipopolysaccharide,and nitric oxide, thereby stimulating EC proliferation (Maier et al., 2004). In addition, Mg^2+^ was able to affect the immunomodulatory properties of MSCs, with increased production of IL-1β and IL-6 by macrophages in MSC medium containing 5 mM Mg^2+^ (da Silva Lima et al., 2018). XU et al.used Mg^2+^-containing hydrogel scaffolds to repair bone defects in rats, and experiments also proved that Mg^2+^ plays an important role in promoting neovascularization and neurogenesis, and has a certain antibacterial effect (Xu et al., 2022). Zhang et al. found that cGRP-mediated crosstalk pathway between peripheral nerve and periosteum-derived stem cells was identified as an important mechanism of Mg-induced bone formation (Zhang et al., 2016). These experiments demonstrated that Mg^2+^ plays an important role in promoting the osteogenic transformation of stem cells.

However, rapid degradation of Mg can have toxic side effects on the cells and tissues surrounding the scaffold, even leading to systemic toxicity. The degradation rate of magnesium can be slowed down by using magnesium alloys, combining magnesium with bioceramics, or surface coating. Wang et al.coated the Mg-strontium scaffold with MAO, SrP, and CaP coatings. By comparison, the CaP coated scaffold exhibited the best corrosion resistance but insufficient osteogenic ability, whereas the SrP coating had superior osteoinductive ability. SrP proved to be a promising coating in terms of adequate degradation rate, osteoinductive properties and beneficial ion release early in healing (Wang et al., 2019).

As a degradable metallic material, Mg can interact with MSCs and exert good osteogenic properties (Zhao et al., 2020). Combined with the price advantage of magnesium, it has great prospects in the repair of bone defects. However, there are still some problems, such as: the pathway of Mg as a cofactor to promote angiogenesis and osteogenesis is not clear, the damage to surrounding tissues caused by the rapid degradation of magnesium and the inability to adapt to the healing speed of new bone.

#### 3.1.4 Zinc

Like magnesium, zinc is also a biodegradable metal. It is the second largest essential trace element in the human body, after iron (Liu et al., 2025). Figure 2d shows a 3D printed porous Zn scaffold. In 2011, Vojtech first systematically investigated the mechanical properties and corrosion resistance of Zn alloys in biological environments (Vojtech et al., 2011). The mechanical strength of pure Zn is relatively low, so researchers usually prepare Zn-based alloy biological scaffolds via 3D printing or pore-forming agents, which not only enhances the mechanical strength of pure Zn scaffolders (yield strength is 90–100 MPa, ductility is 1.2%–2.1%) (Sun, 2022), but also achieves the same purpose as cancellous bone in human body. For example, the mechanical strength of Zn can be greatly improved by adding Li, and the compressive yield strength of Zn-Li alloy with 0.2 wt% content is more than 3 times that of pure Zn. Zn is a degradable metal with a standard corrosion potential of −0.76V, higher than Mg(−2.37 V) but lower than iron(−0.44 V) (Shearier et al., 2016). Unlike magnesium, Zn degradation does not produce hydrogen, which avoids the possibility of local tissue compression and subcutaneous emphysema during the treatment of bone defects with Zn-based scaffolds (Mostaed et al., 2018). In addition, Zn degrades at a slower rate than Mg, closer to the rate of bone defect healing, and the kidney can eliminate the release of excess Zn^2+^, making Zn-based scaffolds safer.

Zinc and zinc-based biological scaffolds have good biocompatibility, bone conduction and osteoinduction properties. Zn^2+^ can stimulate the expression of Runx-2, ALP and OPG and promote the differentiation of osteoblasts (Liu et al., 2025). Xia et al. also found that the culture of BMSCs on porous Zn enhanced the expressions of ALP, Ocn, Osx and Runx-2 (Xia et al., 2023), which may be due to the activation of the Wnt/β-catenin and NF-κB signaling pathways by Zn^2+^ to regulate the production of osteoblasts and osteoclasts, respectively (Wang S et al., 2022). Wang et al. (Wang Z et al., 2022) compared the regulatory ability of pure Zn scaffolds and Zn-Mg alloy scaffolds with different Mg content on osteogenesis and angiogenesis activity. The results showed that both pure Zn and Zn-Mg alloy could promote the proliferation of VECs and reduce the risk of infection in mice. Pure Zn has mild cytotoxicity to cells, while Zn-Sr alloy has better cytocompatibility (Jia et al., 2021).

Zn^2+^ promotes bone regeneration by promoting cell proliferation and differentiation, upregulating the expression of osteogenesis-related genes and proteins, and stimulating angiogenesis. Zn has high corrosion resistance, good biocompatibility and safety. Zn and Zn-based alloys have good bone conduction and osteoinduction ability. In terms of price, zinc is close to magnesium and much lower than titanium and tantalum. However, the shortcomings of pure Zn materials due to insufficient mechanical strength still need to be addressed.

### 3.2 Bioceramic materials

Bioceramic materials are a class of ceramic materials used for specific biological or physiological functions, mainly including HA, tricalcium phosphate(TCP) and bioactive glass(BG), etc. The main components of bioceramic materials are similar to the inorganic composition in human bone, the high content of Ca^2+^ has obvious advantages in promoting new bone formation. Bioceramic materials have good biocompatibility, degradation controllability and good bone conductivity, but there are still some problems such as insufficient mechanical strength, poor cell adhesion and single biological function. SLS technology is suitable for printing high-precision ceramic scaffolds, and ceramic scaffolds can also be fabricated by direct writing with bioinks. Table 2 summarizes the mechanical properties of some bioceramic materials (Liang et al., 2022).

**TABLE 2:** Mechanical properties of bioceramic materials.

Material	Degradability	Density (g/cm^3^)	Modulus of elasticity(GPa)	Compressive strength(MPa)	Yield strength (MPa)
Hydroxyapatite	Inferior	3.18 ∼ 3.41	70∼80	50∼70	50∼150
Tricalcium phosphate	Degradability	3.14	45∼50	20∼25	20∼30
Bioactive glass	Degradability	4.62	70∼80	300∼500	100∼200

#### 3.2.1 Hydroxyapatite

**FIGURE 3:**
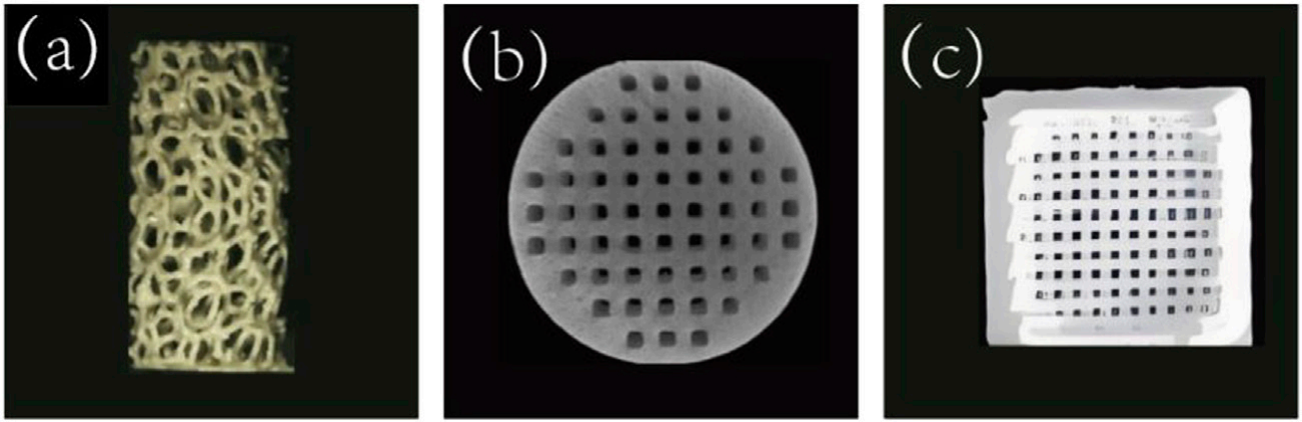
**(a)** Porous HA scaffolds prepared by vat photopolymerization (D'Andrea et al., 2024). Copyright 2023, Wiley. **(b)** TCP scaffolds prepared by suspended surround projection stereolithography(Remy et al., 2021). Copyright 2021, ACS. **(c)** BG scaffolds prepared by 3D printing (Kargozar et al., 2019).

HA, a naturally occurring calcium apatite mineral, is an important inorganic component of human bone (Wang et al., 2024), accounting for approximately 50% of the weight of human bone. Figure 3a shows a porous HA scaffold prepared by vat photopolymerization. The mechanical strength of HA is similar to that of cancellous bone and can resist certain compressive loads. But the tensile strength and brittleness of the material are insufficient (Trzaskowska et al., 2023), and it is fragile under tensile and shear forces. Studies have shown that adding 2wt% carbon nanotubes to HA increases its porosity from about 2.52% to 7.93%. When 1wt% carbon nanotubes were added, the fracture toughness reached 1.88 Mpa m1/2, which was comparable to that of human cancellous bone (Mukherjee et al., 2016). HA is a slightly soluble compound that degrades slowly *in vivo*. This degradation rate is far different from the growth rate of new bone, which is the problem to be solved when HA is used as a biological scaffold to repair bone defects (Erdem et al., 2022).

HA has few adverse reactions after implantation and does not cause immune rejection, so it is relatively safe (Kong et al., 2024). Many studies have shown that HA scaffold has good biocompatibility and osteoconductivity (Liu et al., 2020), but the osteoinductiveness of HA is insufficient, and the antibacterial ability is poor (Lin et al., 2023). Xu et al. compared the effects of bovine-derived HA, pure synthetic HA and COL-I-containing nanoHA in the treatment of rabbit skull defects (Xu et al., 2019). Histomorphological analysis showed that there was always more new bone formation in bovine-derived HA. Russo et al.compared the efficacy of HA/magnetite (90/10 wt%) and HA porous scaffold in the treatment of rabbit femur defect (Russo et al., 2018). By analyzing the volume and mechanical properties of newly formed bone tissue, the good biocompatibility and osteogenic properties of the apatite/magnetite scaffolds were proved, and the bone conduction properties of pure HA were also confirmed. Parisi et al.incorporated sponge into HA to create a HA/sponge scaffold and implant it into rats with tibial bone defects. The results of histological, immunohistochemical and biomechanical analysis showed that the composite could accelerate the degradation of materials and promote the formation of new bone (Parisi et al., 2020).

All of the above studies have shown that HA has good biocompatibility, bone ingrowth potential and a high safety profile. At the same time, it should be noted that the degradation rate of HA is slow, which is much slower than the rate of new bone formation, and the osteoinduction capacity is insufficient.

#### 3.2.2 Tricalcium phosphate

In addition to HA, TCP is also an important inorganic component of human bones and a common bone substitute material. It has good biocompatibility, biodegradability, bone conduction and bone inductivity, among which β-TCP is the most common (Bohner et al., 2020). Figure 3b shows the TCP scaffolds prepared by suspension surround projection stereophotolithography. The calcium-phosphorus ratio of β-TCP is 1.5:1, which is similar to that of normal human bone (1.1–2.1) (Zhang Z et al., 2022). The mechanical properties of β-TCP are affected by a variety of factors, such as pore size and porosity, composite material and scaffold shape. Wang et al.used 3D printing technology to design and prepare β-TCP scaffolds with different pore sizes, among which the β-TCP scaffold with 400 μm pore size has better osteogenic performance (Wang C, 2019). PCL coating improved the mechanical strength and toughness of β-TCP scaffold, and was positively correlated with the concentration of PCL coating. Combined with mechanical properties and microstructure, 0.2 g/mL PCL is the best choice. Vu, etc. designed a cylindrical bone-like scaffold with vertical ridges, horizontal spiral threads, and cylindrical bulges to investigate the effects of surface area and shape on 3D-printed TCP scaffolds (Vu et al., 2021). The results showed that modifying the surface topography of the scaffold by 3D printing could increase the surface area without affecting the mechanical properties, thus improving the biological properties of the scaffold.

β-TCP can promote osteogenic differentiation of MSCs. MSCs cultured on β-TCP showed good biocompatibility, full proliferation, complete proliferation, and maintained osteogenic potential (Chu et al., 2018). This may be due to the activation of calcium signaling pathway by the release of Ca^2+^ from β-TCP, which upregulates the expression of calmodulin and related protein kinases in MSCs (Dai et al., 2024). Porous β-TCP scaffolds with branching channel design can significantly promote the infiltration, migration, proliferation and angiogenesis of human umbilical vein endothelial cells (HUVECs), and promote the proliferation and osteogenic differentiation of human bone marrow stromal cells(hBMSCs) (Qian and Kang, 2024). TCP has good biocompatibility and bone conduction and osteoinduction capabilities, but it is often used to form composite scaffolds with other materials or cytokines for the repair of bone defects. Wong et al. (2021) combined platelet-rich fibrin(PRF) with TCP, in the rabbit model of femur defect, PRF/TCP material was implanted to enhance local bone regeneration of the defect.

The effect of TCP as a biological scaffold in bone defect repair has been verified by a large number of experimental studies, and its effect on MSC and osteoblast is good, which can improve the effect of bone defect repair (Ye et al., 2022). We also need to pay attention to its shortcomings in mechanical properties and degradation rate (Trabelsi et al., 2019), so as to make safer and more efficient biological scaffolds.

#### 3.2.3 Bioactive glass

Unlike HA and TCP, which have higher calcium content, BG contains more SiO_2_. BG was first discovered by Hench in 1969, and it is mainly composed of components such as SiO_2_, Na_2_O, CaO, and P_2_O_5_ (Hench and Wilson, 1984). Figure 3c shows a 3D printed BG scaffold. Later, in 1971, Hench developed 45S5 BG, which has a chemical composition similar to that of human bone and is commonly used as a BG scaffold, in the ratio of 45%SiO_2_, 24.5%Na_2_O, 24.5%CaO, and 6%P_2_O_5_(by weight). When 45S5 BG is implanted into the body, the soluble substances in it are released, and a surface layer of hydrated silica and polycrystalline hydroxycarbonate apatite is formed on the glass, which can enhance the adsorption of growth factors and promote the proliferation and differentiation of osteoblasts (Hench, 2006). The regenerative capacity of BG scaffolds is associated with fabrication methods, scaffold microstructure and porosity characteristics, pre-treatment of the scaffold, and whether growth factors are loaded into the scaffold (El-Rashidy et al., 2017). The elastic modulus and compressive strength of BG scaffolds prepared by rapid prototyping and frozen coagulation are closer to those of human cortical bone.

BG has good cytocompatibility (Simorgh et al., 2022). After dissolving the BG particles in the cell culture medium, osteoblasts were added for culture. The osteoblasts rapidly entered G2 through G1 and S phases, and the cell proliferation rate was significantly accelerated. The growth cycle was about 2 days. This may be because the ions generated after the dissolution of BG, especially silicon ions, can shorten the growth cycle of osteoblasts and promote their proliferation (Sun et al., 2007). A study found that the growth of MSCs on bioglass materials increased calcium deposition and ALP activity, increased the expression of bone-related proteins, and showed higher cell viability (Riva et al., 2023). In addition, BG also promotes the formation of tiny blood vessels. After BG was added to PLA scaffolds, the activity of HUVECs cultured on PLA scaffolds was significantly increased, and the increase in cell viability was best achieved in the PLA-20% BG scaffold (Cichos et al., 2023). Chen et al. prepared a gelatin/SA/58S-BG scaffold based on 58S-BG combined with gelatin and sodium alginate(SA) (Chen, 2023). After the scaffolds were implanted into organisms, the expression of osteogenesis-related factors was increased, and its combined application with extracellular matrix could promote cell adhesion and proliferation, and it also has obvious advantages in osteogenesis and angiogenesis. Additionally, the degradation of BG promotes osteogenic metabolism, induces macrophage M2 polarization, and suppresses local inflammatory responses (Ding et al., 2022).

BG materials have good biocompatibility, among which 45S5 BG has mechanical strength close to human bone and suitable mechanical properties, making it an ideal scaffold material. BG can promote the proliferation and osteogenic differentiation of MSCs, as well as the proliferation of ECs and the formation of new micro blood vessels. However, the preparation of bioactive glass requires strict adherence to the proportions. However, the preparation of bioactive glass requires strict adherence to the proportions, and the exact molecular mechanism by which its ionic products promote osteoblast proliferation and differentiation still needs further research.

### 3.3 Polymer materials

Polymeric materials can be divided into natural polymeric materials and synthetic polymer materials. Natural polymeric materials mainly include gelatin, chitosan(CS), alginate(ALG), cellulose, etc. Natural polymers are derived from living organisms and have a structure similar to that of the human extracellular matrix (Kuna et al., 2024). They can reduce immune rejection and promote cell adhesion, proliferation and differentiation. Their degradation rate can be regulated by modification, and the degradation products can be metabolised and absorbed by the body. In addition, some natural polymers are biologically active. However, natural polymers have low mechanical strength and are difficult to bear weight alone. The degradation rate of some materials may be too fast to match the rate of new bone formation (Bao et al., 2019). Some natural polymers may retain xenoantigens, presenting a risk of immunogenicity. Natural polymers have poor resistance to high temperatures, limiting the application of certain processing techniques. Synthetic polymer materials mainly include PCL, PLA, polylactic acid-glycolic acid, etc. Its advantages are good biocompatibility and biodegradability, less adverse reactions and high safety. In addition, the characteristics of porosity, hydrophilicity, and swelling also provide more opportunities for it to be used as a carrier for repairing bone defects. The interaction of polymer materials with MSCs, osteoblasts and ECs contributes to the formation of new bone and blood vessels. However, the mechanical properties of polymer materials are suboptimal and the degradation rate is difficult to control, which may be the biggest challenge in their application. FDM is suitable for printing thermoplastic polymers such as PLA and PCL, while stereolithography and digital light processing techniques are suitable for printing photosensitive resins with high resolution. Table 3 summarizes the mechanical properties of some polymer materials. Table 4 summarizes the biological properties of some biological scaffold materials and their interactions with cells.

**TABLE 3:** Mechanical properties of polymer materials.

Material	Provenance	Degradation product	Density (g/cm^3^)	Young’s modulus (GPa)	Yield strength (MPa)	Tensile strength (MPa)
Chitosan	Crustaceans	Glucosamine	1.75	18.8 ± 1.5	30–80	50–100
Alginate (sodium)	Algae and bacteria	Monosaccharides or oligosaccharides	1.0	0.1–10	30–50	/
Silk fibroin	Silkworm cocoon	Amino acids and peptides	0.622	10–17	100–150	/
Polylactic acid	Lactic acid	CO_2_ and H_2_O	1.25 ∼1.28	3–4	50–70	40–70
Polycaprolactone	ε-caprolactone	CO_2_ and H_2_O	1.146	/	10-25	20–45

Note: “/” indicates that clear data has not been found yet.

**TABLE 4:** Biological properties of bone tissue engineering scaffold materials.

Material	Bone inductance	Effects on mesenchymal stem cells	Effect on osteoblasts	Effect on endothelial cells	Antibacterial property
Porous titanium	−Dou et al. (2019)	-	-	-	−Li (2019)
Porous Tantalum	+Wang et al. (2023c)	Activate MAPK/ERK signaling pathway to promote osteogenic differentiation	Promote cell adhesion and proliferation	Enhance the formation of capillary-like vascular networks by cells	−XU (2023)
Magnesia	+Zhang Y et al. (2022)	Promote cell proliferation, regulate macrophage production of IL-1 β and IL-6	Promote cell adhesion, proliferation, and mineralization. Inhibit the biological activity of osteoclasts	Promote the synthesis of angiogenic factors and stimulate cell proliferation	+Zhang Y et al. (2022)
Zinc	+Zhao and Chen (2024)	Activate Wnt/β - catenin, PI3K/Akt, and MAPK/Erk signaling pathways to promote osteogenic differentiation	Stimulate the expression of transcription factors Runx-2, ALP, and OPG genes	Promote cell proliferation and angiogenesis	+Zhao and Chen (2024)
Hydroxyapatite	−Xu et al. (2019)	-	-	-	−Lin et al. (2023)
Tricalcium phosphate	+Zhang Z et al. (2022)	Activate the calcium signaling pathway and upregulate the expression of calmodulin	Promote cell adhesion, proliferation, and extracellular matrix formation	Promote cell infiltration, migration, proliferation, and angiogenesis	−GAO and LIU (2022)
Bioactive glass	+Wu J et al. (2022)	Enhance cell vitality and promote osteogenic differentiation of cells	Shorten the growth cycle of cells and promote their proliferation	Enhance cell vitality and promote small vessel formation	−Fernandes et al. (2017)
Chitosan	+Yadav et al. (2021)	Elevated expression of ALP, COL-I, osteocalcin, and BMP-4	-	Promote cell migration	−Tang et al. (2024)
Alginate	−Wang Z et al. (2022)	-	-	-	−Xie (2022)
Silk Fibroin	+Wu H et al. (2022)	Elevated expression of osteocalcin, RunX 2, and CD 29/CD 44	-	Activate integrin/PI3K/Akt and glycolysis signaling pathways to accelerate angiogenesis	−Pan et al. (2023)
Polylactic acid	−Alavi, et al. (2023)	-	-	-	−Liu and Zheng (2024)
Polycaprolactone	−Park et al. (2021)	-	-	-	+Hajduga et al. (2022)

Note: “+” represents existence; “−” represents non-existent or the effect is not obvious.

#### 3.3.1 Chitosan

**FIGURE 4:**
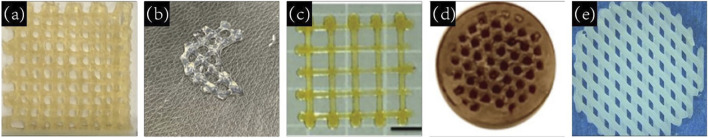
**(a)** 3D printed CS scaffold (Sadeghianmaryan et al., 2020).Copyright 2020, Elsevier. **(b)** 3D printed ALG gel scaffold (Chawla et al., 2020). Copyright 2020, Elsevier. **(c)** 3D printed silk fibroin (SF) scaffolds (Mu et al., 2020). Copyright 2020, MDPI. **(d)** PLA scaffolds prepared by FDM by alizarin red staining (Diez-Escudero et al., 2021).Copyright 2021, Elsevier. **(e)** SEM image of a 3D printed PCL scaffold (Rashad et al., 2018). Copyright 2018, America Chemical Society.

CS is a degradable natural polymer derived from the deacetylation of chitin, which is widely available and inexpensive, and is commonly found in the shells of crustaceans such as shrimp and crabs (Mallakpour and Khadem, 2018). Figure 4a shows a 3D printed CS scaffold. CS has good biocompatibility due to its structure and composition being very similar to glycosaminoglycans (Yadav et al., 2021). However, the disadvantages of low mechanical strength and rapid degradation rate of CS have also become obstacles to its application. The swelling capacity is an important parameter of CS materials, which is related to the water-holding capacity, nutrient transport ability, cell infiltration and degradation rate of CS scaffolds. The pore size and porosity are closely related to the swelling capacity of scaffolds. When the pore size and porosity increase, the swelling capacity and degradation rate of scaffolds also increase.

BMSCs exhibited significant osteogenic differentiation ability on CS-based scaffolds,the expression of ALP, COL-I, OCN and BMP-4 were significantly increased after culturing on CS scaffolds (Midha et al., 2021). However, the adhesion and proliferation ability of MSC on the surface of chitosan hydrogel is defective, and the adhesion and proliferation ability is significantly enhanced after deacetylation (Ding et al., 2016), adding a certain concentration of arginine-glycine-aspartic acid short peptide into CS-based hydrogels could also promote the adhesion of MSCs and maintain cell activity (Wang, 2018). CS hydrogels also promoted the migration of ECs and had obvious chemotaxis. The co-culture of ECs and smooth muscle cells(SMC) was beneficial to the vascularization of ECs (Wang, 2018). The primary amino group in CS has the functions of controlling drug release, mucosal adhesion, *in situ* gelation and transfection, which makes CS scaffold can be used as a carrier to release drugs and cytokines into the bone defect area (Bharathi et al., 2022). Xue et al. added simvastatin to the CS scaffold, after adding 4 mg simvastatin, cell proliferation was better and ALP significantly increased (Xue, 2019). Moreover, in the rat skull defect model, the addition of 4 mg simvastatin resulted in earlier and more significant bone-like tissue formation. Chen et al. added particles containing BMP4 and basic fibroblast growth factor (FGF2) to CS, and the repair effect was good in the rabbit bilateral radius defect model, which could withstand the maximum lateral stress standard and had a high degree of mineralization of newly formed bone (Chen, 2020).

CS-based scaffolds can significantly improve the osteogenic effect of MSCs and promote the vascularization of ECs.Although adjusting the pore size and porosity of the stent (Lekhavadhani et al., 2023) or adding synthetic polymers (Sun et al., 2022) and metallic materials (Wang S, 2019) can improve the mechanical strength of CS scaffolds to a certain extent,but further research is needed. CS materials have good biocompatibility and can promote new bone formation and mineralization by stimulating osteogenic genes. As drug carriers and fillers for bone defects, CS materials have great prospects in bone defect applications.

#### 3.3.2 Alginate

Similar to CS, ALG is also a biodegradable natural polymer mainly found in seaweed and bacteria (Rastogi and Kandasubramanian, 2019). Figure 4b shows a 3D printed ALG gel scaffold. SA is soluble and the chelate formed by ALG and divalent metal cation is gelled (Fan et al., 2022). Similar to most hydrogels, ALG has good biocompatibility but poor mechanical properties (Hurtado et al., 2022). The poor adhesion between cells and ALG may be due to the lack of structural domains that bind to cells (Zia et al., 2015). Blending ALG with other materials can improve the problems of poor cell adhesion, insufficient antibacterial ability (Xie, 2022) and poor mechanical properties of ALG hydrogel (Hu et al., 2023). Arslan et al.added triacetin(TA) and tributyl citrate(TBC) to ALG by solvent casting technique. After TA and TBC were added, the pore size of the scaffold decreased slightly, and the modification of TA and TBC reduced the swelling rate of the scaffold and significantly accelerated the degradation rate of the scaffold. The mechanical test results showed that TA and TBC increased the tensile stress and fracture elongation of the bracket (Arslan et al., 2023). In order to improve the mechanical strength and osteogenesis of ALG scaffolds, Silva-Barros found that LG increased the mechanical strength of SA-based scaffolds, resulting in compressive strength and Young’s modulus values within the range of bone trabeculae. Especially when LG:SA was 1:2, the scaffold strength showed an increase of about 15%. Moreover, the scaffold has great cytocompatibility and can promote the adhesion and proliferation of osteoblasts (Silva-Barroso et al., 2023).

ALG is frequently combined with other materials to enhance mechanical strength and serve as a platform for the delivery and promotion of bone repair materials for bone defect repair. Wang C et al., 2022 developed a system comprising osteoblasts, calcium ALG scaffolds, and a self-constructed bioreactor system. The calcium ALG scaffold has been demonstrated to promote the growth and differentiation of human bone cell clusters, retain cell proliferation ability and vitality, and upregulate the expression of bone-related genes and the formation of biological apatite crystals (Chen et al., 2015). Westhrin et al. cultured MSCs on mineralized alginate gel scaffolds and observed high activity and metabolic activity. The mRNA levels of osteoblast-specific genes expressed by MSCs were significantly increased, providing a favorable environment for enhanced osteogenic differentiation (Westhrin et al., 2015).

Similar to the majority of hydrogels, ALG exhibits favourable biocompatibility and is readily obtainable, which are the advantages of ALG as biological scaffolds. Nevertheless, the impact of ALG scaffolds on MSCs remains inconclusive, and the shortcomings associated with insufficient mechanical strength and challenging degradation further restrict the utilisation of ALG materials.

#### 3.3.3 Silk fibroin

SF is a naturally biodegradable protein polymer extracted from the cocoon of the silkworm and widely available in nature (Rockwood et al., 2011). Figure 4c illustrates a three-dimensionally printed SF scaffold. SF exhibits excellent biocompatibility, controllable degradation rate, non-toxic degradation byproducts, and a low likelihood of inducing inflammatory responses (Wang et al., 2015). According to the specific requirements of the intended application, SF can be processed into a variety of forms, including microspheres, films, and scaffolds, among others (Li and Xie, 2023). Deshpande et al. prepared SF microparticles that were non-cytotoxic and did not cause irritation, inflammation, or allergic reactions. The subcutaneous implantation of SF microparticles scaffolds has been demonstrated to result in significant absorption, promotion of fibroblast infiltration and the number of new vessels, and the formation of good tissue integration (Deshpande et al., 2022).

SF has been demonstrated to exert a certain osteogenic effect, which can be observed in the enhanced expression of osteogenic factors, including ALP, Runx-2, COL-I and OCN, amongst others. RNA sequencing and proteomic analysis have revealed that SF enhances the angiogenic and immunomodulatory effects of MSCs by activating integrin/PI3K/Akt and glycolysis signalling pathways (Zhang et al., 2023). Furthermore, SF scaffolds have been demonstrated to promote osteogenic differentiation and mineralisation of adherent hBMSCs, as evidenced by the elevated expression of Runx-2, OCN and CD29/CD44, in addition to the assessment of glycosaminoglycan and alizarin red staining (Yan et al., 2019). The co-culture of induced endothelial cells derived from MSCs and MSCs on a SF protein scaffold has been demonstrated to enhance the osteogenic differentiation of MSCs, with significantly increased ALP levels and calcium deposition. However, it has also been shown that simple SF scaffolds lack the capacity to fully regenerate large bone defects (Wu H et al., 2022). It is often necessary to pre-inoculate with undifferentiated stem cells or add other materials with osteogenic properties prior to implantation. Gao et al. prepared SF/nano-HA composite scaffolds with varying contents of porous SF as the main body. The findings demonstrated that SF/nHA scaffolds exhibited robust adhesion with BMSCs, accompanied by a notable elevation in DNA content, ALP, calcium content, Runx-2, and OCN expression. Among these, SF-20HA exhibited the most pronounced osteogenic induction capacity. The results of implantation in a rat skull defect model further substantiated that the SF-20HA scaffold elicited the most efficacious repair of rat skull bone defects (Gao, 2020).

SF is an FDA-approved biosafety material with favourable biocompatibility and biodegradability. Its interaction with MSCs and osteoblasts contributes to osteogenic differentiation. SF based biomaterials have been extensively studied in the field of cartilage/osteochondral repair (Zhou et al., 2022). However, as a natural biological material, SF has insufficient mechanical strength and lacks antibacterial properties (Pan et al., 2023). Given its favourable biocompatibility and osteoinductivity, SF is anticipated to emerge as a pivotal material in the repair of bone defects.

#### 3.3.4 Polylactic acid

Unlike previous natural polymers, PLA is a biodegradable polymer formed by the polymerization of lactic acid (Song et al., 2020). Researchers have conducted in-depth research on it and have produced it on a large scale (Pesaranhajiabbas et al., 2023). Figure 4d illustrates the PLA scaffold prepared by FDM with alizarin red staining. Due to its solid state and non-toxic degradation products, PLA does not cause adverse reactions in the human body and is highly biocompatible, making it an ideal implant material for the human body (Singhvi et al., 2019). PLA has an appropriate mechanical strength and elastic modulus similar to human bone. However, pure PLA material has disadvantages, including a low degradation rate, poor cell adhesion, an absence of antibacterial properties (Liu and Zheng, 2024), poor bone conduction and osteogenic performance (Alavi et al., 2023). To overcome these problems, PLA is often combined with other biomaterials before application. For instance, the addition of bioglass 45S5BG to PLA results in the uniform distribution of BG particles within the PLA matrix, thereby enhancing the mechanical strength of the scaffold by up to 80% (Sultan et al., 2022). Ding et al. employed 3D printing technology to fabricate PLA scaffolds with varying pore sizes (500 μm, 750 μm, and 1000 μm). The scaffolds with smaller pores exhibited higher The compression modulus was found to be comparable to that of cancellous bone. Simple PLA scaffolds were observed to exhibit good biocompatibility, and 750 μm pore size PLA scaffolds were identified as a suitable bone substitute for the repair of large bone defects (Ding et al., 2022).

3D printed PLA scaffold is a feasible choice for BMSC culture and osteogenic differentiation (Liu et al., 2023). The porous structure of the PLA scaffold can facilitate the orderly crawling and growth of bone cells. The culturing of BMSCs on scaffolds prepared with an 8% PLC solution allows for the provision of additional space, which in turn facilitates the migration, adhesion, and proliferation of cells into the internal pore structure of PLA scaffolds. Furthermore, in a rat model of osteochondral defect, the regeneration ability of the defect is enhanced following the implantation of the scaffold (Wang Z et al., 2022; Zhao et al., 2022). In order to optimise the osteogenic performance of PLA-based scaffolds, numerous scholars have conducted experiments. Yao et al. conducted a comparative analysis of freeze-dried PLA scaffolds, 3D-printed PLA scaffolds, and 3D-PLA-BMP-2 scaffolds with BMP-2, and subsequently implanted them into the bilateral femoral condyles of rabbits. The micro-CT results demonstrated that the bone repair rate, trabecular volume, and trabecular thickness of the 3D-PLA-BMP-2 group were significantly higher than those of the other two groups (Yao et al., 2020). Furthermore, additional experiments have corroborated the excellent biocompatibility, osteoconductivity, and osteoinductivity of the 3D-PLA-BMP-2 scaffold.

PLA exhibits excellent biocompatibility, non-toxicity and suitable mechanical properties, rendering it a commonly utilised scaffold material. However, pure PLA material displays inadequate osteogenic properties. When combined with materials that possess osteogenic capabilities, it can more effectively facilitate the repair of bone defects.

#### 3.3.5 Polycaprolactone

PCL is also a high molecular weight polymer, which is an aliphatic polyester composed of repeated units of caproate (Arif et al., 2022). Figure 4e illustrates a SEM image of a 3D printed PCL scaffold. Multiple methods can be used to prepare PCL porous scaffolds, including 3D printing, freeze-drying, electrostatic spinning, salt immersion, etc. (Siddiqui et al., 2021). Koch et al. conducted a comprehensive assessment of a PCL scaffold prepared via FDM technology. The compression modulus of the pure PCL scaffold was found to be 6 MPa, which is comparable to that of human cancellous bone. Following the addition of hydrogel and subsequent printing, the compression modulus of the scaffold decreased to approximately 4 MPa (Koch et al., 2022). However, there is a notable discrepancy between the elastic modulus and compressive strength of PCL and those of human cortical bone, and its hydrophilicity is relatively poor. A number of studies have been conducted with the aim of addressing these issues, including the addition of Mg_3_(PO_4_)_2_ to PCL scaffolds. This has resulted in an effective improvement in the compressive strength of the scaffold material, accompanied by a gradual decrease in the contact angle and an enhancement in the hydrophilicity of the scaffold as the Mg_3_(PO_4_)_2_ content increases. Furthermore, the dissolution of Mg_3_(PO_4_)_2_ on the surface of the scaffold increased the contact area between PCL and lipase, thereby accelerating the degradation rate of the scaffold (Lei, 2022).

PCL scaffolds have been demonstrated to possess a limited capacity to facilitate cell attachment, proliferation and differentiation. Consequently, they are frequently combined with other materials in the preparation of biological scaffolds, with the objective of enhancing the interaction between the scaffolds and osteoblasts, augmenting the mechanical strength and bone induction capacity of the scaffolds. Park et al. employed 3D printing technology to fabricate a PCL/T50 scaffold with a PCL:β-TCP ratio of 1:1, and observed that in comparison with a PCL scaffold, the PCL/T50 scaffold markedly augmented the volume of new bone formation (Park et al., 2021). Heo et al. isolated fish bone extract containing tripeptide and coated it on the surface of a PCL scaffold prepared by 3D printing. The expression of osteogenic markers, including ALP, osteopontin, OCN and BMP-2, was significantly increased in cell proliferation and osteogenesis experiments, and the proliferation and calcium deposition rate of osteoblasts were significantly accelerated (Heo et al., 2019). Additionally, the addition of CaCO_3_ to PCL scaffolds has been demonstrated to promote the formation of new bone and blood vessels (Saveleva et al., 2021).

PCL is applied in medical fields such as tissue engineering and drug delivery (Rahimkhoei et al., 2023). The complete degradation of PCL results in the production of CO_2_ and H_2_O, which are safe and non-toxic *in vivo* and possess antibacterial properties (Hajduga et al., 2022). In addition, PCL exhibits favourable cell compatibility, processability, and mechanical properties, which are advantageous for its use as a biological scaffold material (Labet and Thielemans, 2009). However, PCL also presents certain disadvantages, including a slow degradation rate within the human body, poor hydrophilicity, limited cell adhesion, weak capacity to promote cell proliferation and differentiation, and poor bone induction ability. Table 4 provides a summary of the biological properties of various biological scaffold materials and their interactions with cells. Table 5 summarizes the representative reviewed research articles in each section.

**TABLE 5:** Biological scaffolds for bone-related disease management and tissue regeneration.

Material	Tested Cells	*In Vitro* Results	Animal Model	*In Vivo* Results	References
TI6AL4V	——	——	rabbit tibial defect model	Porous titanium alloy implants show potential for bone defect reconstruction	Jabbari et al. (2023)
pTa	BMSCs	pTa promotes BMSC adhesion, growth, and osteogenic differentiation	——	——	Dou et al. (2019)
PCL/Zn	MC3T3-E1	PCL/Zn scaffolds exhibit excellent mechanical properties and cytocompatibility	Rat calvarial defect model	Osteogenic effects of PCL/Zn scaffolds increase with Zn content (0–2 wt%)	Wang S et al. (2022)
HA/SPG	——	——	Rat tibial bone defect	Accelerates material degradation and enhances new bone formation	Parisi et al. (2020)
β-TCP	HBMSCs, HUVECs	Promotes HUVEC infiltration, migration, proliferation, angiogenesis, and hBMSC osteogenic differentiation	Mouse subcutaneous	Promotes rapid vascularization and stimulates osteocyte recruitment	Qian and Kang, (2024)
Bioglass	Osteoblasts	Enhances osteoblast proliferation	——	——	Sun et al. (2007)
Alginate	MSC	Induces hMSC osteogenic differentiation and accelerates mineralization	——	——	Papaccio et al. (2015)
PLA, PLA–BMP-2	——	——	Rabbit bilateral femoral condyle defects	Significantly improves bone repair rate, bone mass, and trabecular thickness	Yao et al. (2020)
PCL/β-TCP	——	——	Canine mandibular defect model	Facilitates rhBMP-2 delivery and maintains space for bone formation in mandibular defects	Park et al. (2020)

### 3.4 Hybrid biomaterials

Hybrid bioscaffolds are composite scaffolds that combine two or more materials. The purpose is to overcome the limitations of a single material through synergistic effects between materials, in order to optimize the effectiveness of tissue regeneration. The core design concept is to integrate the physical, chemical and biological properties of different materials to enhance the mechanical properties, biocompatibility and functionality of the scaffold. Hybrid bioscaffolds achieve complementary functions through multi-material combinations. Common forms include: natural-synthetic composites, such as collagen combined with PLA, which combines the bioactivity of natural materials with the mechanical strength of synthetic materials. Organic-inorganic material composites, such as Hydroxyapatite composite with polycaprolactone, simulate the mineral-organic matrix structure of natural bone. Dynamically responsive material combinations, such as temperature-sensitive hydrogel combined with metal nanoparticles, to realize the 4D function of the scaffold.

The inter-combination between polymers, ceramics, and metals is also a common form. Ma et al. successfully fabricated a composite scaffold with high strength, superelasticity and bioactivity by introducing urethane-based PEGylated poly (glycerol sebacate) (PEGSU) in β-TCP. The scaffold exhibits excellent mechanical properties and biological functions by simulating the properties of polymer-ceramic composite materials as well as the room temperature self-supporting mechanism. In a rabbit cranial defect model, the scaffold demonstrated a significant effect on the repair of critical-sized bone defects compared to the β-TCP scaffold alone, with significantly higher new bone volume and trabecular thickness (Ma et al., 2021).

The main challenges faced by polymer scaffolds in clinical applications are their insufficient mechanical strength and mismatched degradation rates. To address this issue, the introduction of metallic materials into polymer scaffolds has been shown to be an effective strategy (Mohammadi Zerankeshi et al., 2022). Experiments have shown that doping bronze particles can increase the elastic modulus of pure PLA samples printed in the 0°and 90° directions by 10% and 27%, respectively (Alam et al., 2020). Adding more Ti can continuously increase the compressive and tensile strength of PLA/Ti composite materials until 10 vol% is added (Lee et al., 2019). Furthermore, the incorporation of metal fillers could enhance cell viability, promote osteogenic differentiation, enhance angiogenesis, and improve antimicrobial properties.

Pure ceramic materials are highly brittle and prone to fracture, often requiring composite design to improve toughness. The combination of ceramics and metals is an effective way (Sun et al., 2024). Many ceramic materials have significant bone conductivity but lack osteoinductivity. The combination with metal materials can dynamically regulate the degradation rate and promote osteogenic differentiation. Magnesium-containing calcium phosphate cement demonstrates sustained magnesium release, providing long-term mechanical stability and osteogenic effects while exhibiting no cytotoxic effects on hBMMSCs and macrophages (Wu J et al., 2022). Incorporating HA into pure Zn is an effective method that can adjust its degradation rate and improve its biocompatibility *in vitro* and *in vivo*. In contrast to pure Zn, Zn-5HA composite materials exhibit better performance in osteogenesis (Yang et al., 2018).

In conclusion, hybrid bioscaffolds significantly enhance the effectiveness and safety of tissue regeneration through material synergy and structural innovation. It can regulate the balance of strength and toughness, modulate the degradation rate, and promote cell adhesion and vascularization. However, it is also important to consider the insufficient bonding strength of different materials that may lead to interface peeling and the safety of complex scaffolds in the *in vivo* microenvironment.

## 4 Factors affecting bone defect repair

### 4.1 Pore size and porosity

The pore size and porosity of scaffolds affect the proliferation and ingrowth of cells, and the design of a scaffold with appropriate structure is conducive to the early formation of new bone tissue. This may be due to: 1. The compressive strength and elastic modulus of the scaffold decrease with the increase of porosity, and the porous structure significantly reduces the stress shielding effect. 2. Large pore size facilitates the transport of oxygen and nutrients, thereby promoting cell proliferation, differentiation, intercellular signaling, and angiogenesis. 3. The increase of pore size or porosity can reduce the contact angle, and the relatively higher surface roughness shows a more hydrophilic surface, thus improving the adsorption capacity of proteins. However, too large or too small pore size and porosity can have certain negative effects. The velocity of cells passing through the center of the pore increased significantly with the increase of the pore size, resulting in vortex formation and energy dissipation in the center of the scaffold, which may affect the cell inoculation of the scaffold (Wang et al., 2023a). For different materials, the appropriate porosity and pore size can vary, taking into account factors such as mechanical strength and degradation rate. Chen et al. fabricated porous Ti6Al4V scaffolds by SLM and found that smaller pore size and porosity led to better cell adhesion, proliferation and osteogenic differentiation of rBMSCs, especially scaffolds with pore size of 500 μm and porosity of 60% showed the best results, which was more conducive to bone inward growth (Chen et al., 2020). Luo et al. fabricated porous tantalum scaffolds based on SLM, and confirmed that porous Ta scaffolds with pore size of 400–600 μm and porosity of 75% are beneficial to osteogenesis and bone integration, and have great potential in bone defect repair (Luo et al., 2021). Some studies have also shown that porous tantalum scaffolds with pore size of 500 μm and porosity of 70% can upregulate the expression of osteogenesis-related genes and show the highest bone growth area and bone contact rate *in vivo* (Wang et al., 2023b). In addition, zinc and magnesium are also commonly used metal scaffold materials. Wang et al. found that zinc-magnesium alloy scaffolds with a porosity of 60% and a unit size of 2.5 mm or a porosity of 80% and a unit size of 2 mm had the best osteogenic ability (Wang et al., 2023c). In addition to metal scaffolds, there are also many studies on the pore size and porosity of bioceramic scaffolds. Qin et al. studied the effect of calcium phosphate scaffolds with different pore sizes on bone defect repair, and found that calcium phosphate scaffolds with porosity of 70% and pore size of 0.8 mm had superior advantages for initial bone formation and maturation, and total bone formation was the largest (Liu et al., 2019). For calcium silicate scaffolds with porosity of 58%, pore size of 600 μm can guide the inward growth of new bone and accelerate bone regeneration and repair better (Qin et al., 2022). For chitosan scaffolds, a pore size of 100–300 μm is an ideal choice for osteogenesis, but a pore size of 400 μm has a strong ability to promote angiogenesis during osteogenesis (Lekhavadhani et al., 2023). In conclusion, most materials can play a better role in repairing bone defects when the pore size is 400–600 μm and the porosity is about 70%, and there are slight differences between different materials. The effects of pore size and porosity should also be taken into account when designing scaffolds using 3D printing technology.

### 4.2 Degradation behavior

Many biological scaffold materials can be degraded *in vivo*, such as magnesium and zinc in metal materials; tricalcium phosphate and bioactive glass in bioceramic materials; Chitosan and silk fibroin in synthetic materials, etc. The slow degradation of scaffolds *in vivo* changes the concentration of surrounding ions and provides an environment conducive to bone defect repair. Magnesium degradation in the body can promote the deposition of calcium and phosphorus, which are converted into bone tissue, and magnesium can also be used as a cofactor to promote the formation of new bone. The release of Ca^2+^ from β-TCP activates the calcium signaling pathway, which in turn upregulates the expression of calmodulin and related protein kinases in MSCs (Dai et al., 2024). The degradation behavior is affected by many factors, including crystallinity, environmental PH, temperature, etc., but it is mainly related to the specific surface area of the scaffold, which increases with the increase of porosity. Scaffolds with larger specific surface area have more contact areas with body fluids, and the degradation rate is faster. Saad et al. compared the degradation behaviors of porous magnesium alloys with porosity of 30%, 41% and 55% in dynamic simulation of body fluids, and the results showed that the degradation rate of porous magnesium alloys with porosity of 41% was more appropriate *in vivo* (MdSaad et al., 2019). Too fast or too slow degradation may affect the repair of bone defects, change the mechanical strength of scaffolds, and fail to play an effective supporting role. For example, pure magnesium scaffolds degrade rapidly, causing damage to surrounding tissues and failing to adapt to the healing speed of new bone (Lu et al., 2017). HA is a microsoluble compound that degrades slowly *in vivo*. Implanted porous HA cylinders into cancellous bone of rabbits, only 5.4% volume reduction was observed after 6 months, while the volume reduction of TCP was 85.4% under the same conditions (Wang and Yeung, 2017). The degradation rate of ALG is slow or even difficult to degrade, which may be due to the lack of ALG-degrading enzymes in human body (Li et al., 2023). It is important to note that, unlike *in vitro* experiments, the concentration of ions around scaffolds after degradation *in vivo* tends to be slightly lower than *in vitro* due to the circulation of body fluids, and the rate of degradation also varies. PCL degrades quickly in the natural environment, but slowly in the body, taking 6 months to 2 years (Arif et al., 2022). By setting the pore size and porosity of the scaffold by 3D printing technology, or by modifying the surface of the scaffold and adding other materials, the degradation rate of the scaffold can be adjusted within a certain range and the ability of bone defect repair can be improved.

### 4.3 Osteogenic activity

**FIGURE 5:**
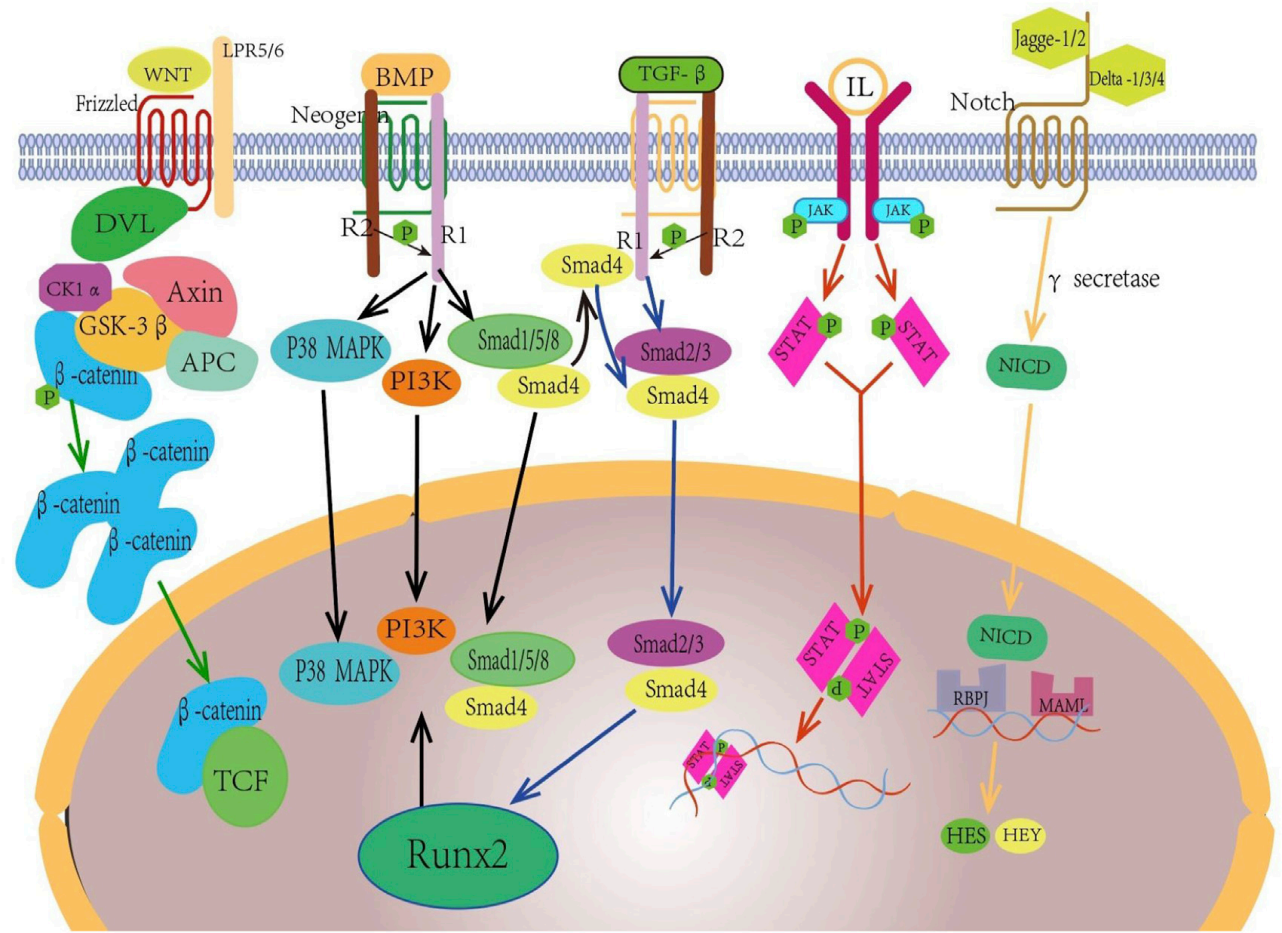
Wnt/β - catenin, Bmp/Smad, TGF - β/Smad, JAK/STAT, North and other pathways play important roles in promoting the generation of new bone tissue.

**FIGURE 6:**
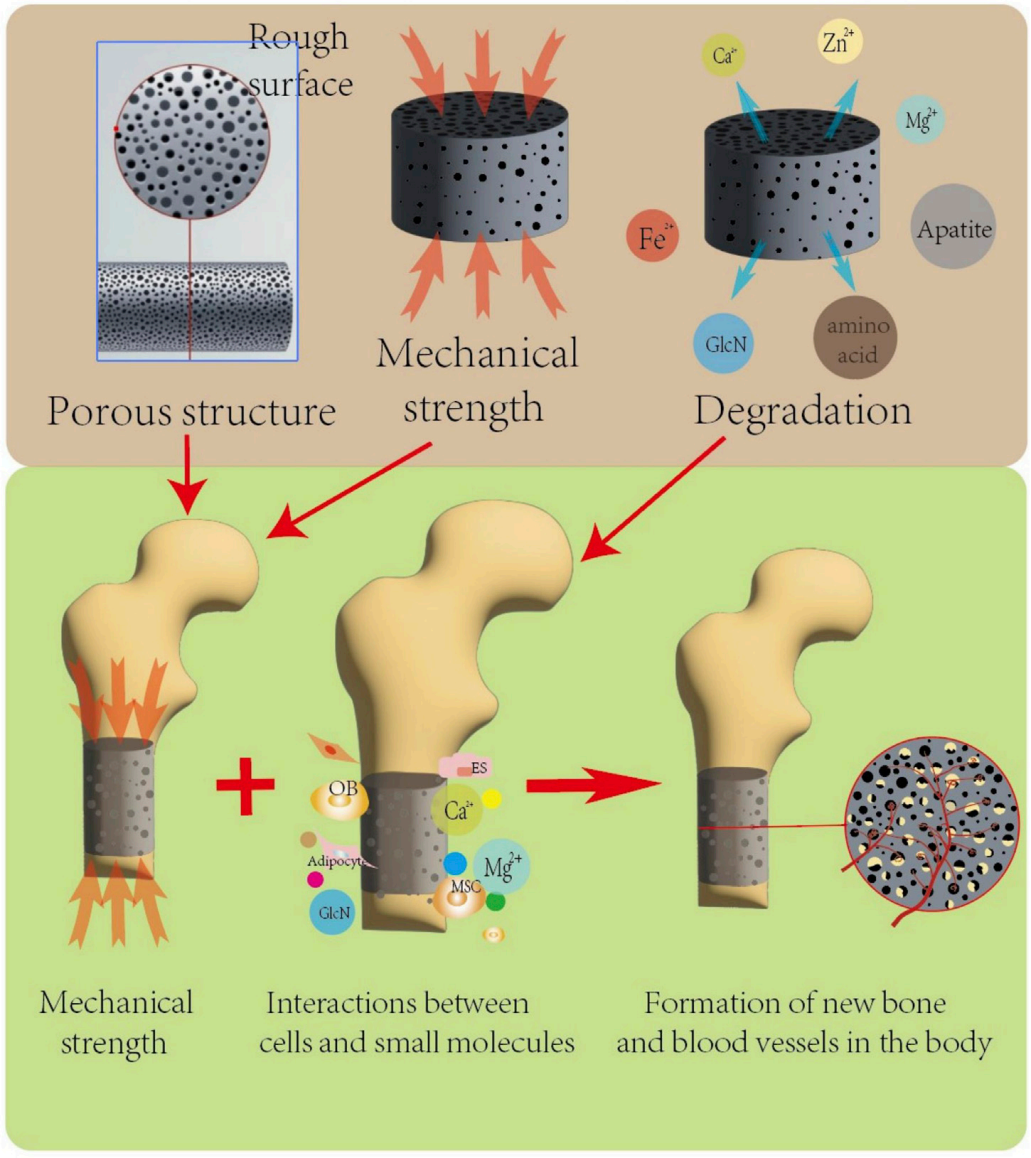
The porous structure and mechanical strength of the scaffold affect its structural stability under compression *in vivo*, and the porous structure is conducive to the growth of new bone tissue and blood vessels. The degradation products of biodegradable scaffolds can provide a favorable local microenvironment for bone defect repair at the transplant site, and multiple factors work together to promote the formation of new bone and blood vessels.

*In vivo* osteogenesis is affected by many factors, and scaffolds with different structural designs show different osteogenic abilities due to their different physical and chemical properties. The physical factors are mainly elastic modulus, compressive yield strength, pore size, porosity and degradation rate of the scaffold. On the one hand, the implanted scaffold should be close to the mechanical strength of the bone at the defect to provide sufficient support capacity and reduce the influence of stress shielding effect. On the other hand, the scaffold needs to provide space and environment for the inward growth of new bone. Chemical and biological factors are also important factors affecting bone defect repair, mainly related to the material of the scaffold. Bone tissue grows and repairs under the action of various cells and cytokines such as osteocytes, MSCs, osteoblasts, osteoclasts, and ECs, and functions through TGF-β/bmp, TGF-β1/Smad, Wnt/β-catenin, PI3K/AKT/GSK3β/β-catenin, Notch (Chen et al., 2012; Zhang et al., 2020; Zhao et al., 2020; Ballhause et al., 2021; Yuan et al., 2023) and other signaling pathways. Figure 5 is a schematic diagram of some common regulatory pathways affecting bone growth. The expression of some osteogenic related factors, such as alkaline phosphatase(ALP), type I collagen(COL-I), osteocalcin(OCN), and RUNX-2, also plays an important role in the process of bone repair. After some materials are degraded *in vivo*, some scaffolds are gradually degraded *in vivo*, and the released ions can have an impact on MSCs, osteoblasts and ECs, and promote osteogenic differentiation and the formation of new capillaries by activating related signaling pathways. For example, zinc-containing scaffolds can form a local high Zn^2+^ environment during degradation, and promote the osteogenic differentiation of MSCs by activating the wnt/β-catenin, PI3K/Akt, and MAPK/Erk signaling pathways (Jia et al., 2021; Wang S et al., 2022). Silk fibroin-containing scaffolds can enhance the expression of RunX2, osteocalcin, and CD29/CD44, promote osteogenic differentiation and mineralization, and promote neoangiogenesis by activating integrin/PI3K/Akt and glycolytic signaling pathways (Yan et al., 2019; Zhang et al., 2023). In addition, some materials have excellent antibacterial properties and play an important role in promoting bone tissue growth. Yuan et al. (2020) added nanosilver(NSAg) to the β-TCP scaffold, and the NSAg-TCP material inhibited the growth of *Staphylococcus aureus* and *Escherichia coli* and was non-cytotoxic to human BMSCs.The study of He et al.also proved that Ag/Co/β-TCP materials exhibited good biocompatibility and antimicrobial properties (He et al., 2022). In conclusion, osteogenic capacity is also a must consideration when selecting and preparing biological scaffolds. Figure 6 Schematic diagram of several factors affecting the repair effect of bone defects.

### 4.4 Other factors

In addition to the aforementioned factors, numerous other aspects of biological scaffolds can influence the effectiveness of bone defect repair, such as mechanical properties, rheological characteristics, material modifications, surface modifications, and biocompatibility.

Different skeletal and cartilage tissues are subjected to varying mechanical loads. When scaffolds are implanted into cancellous bone, the required compressive strength ranges from 2 to 12 MPa, whereas for trabecular bone, it increases to 45–130 MPa (Ingole et al., 2019). By selecting appropriate material combinations and structural designs to control porosity and compressive strength, scaffolds can provide a mechanical environment similar to native tissue, facilitating cellular differentiation. For instance, α-calcium sulfate hemihydrate(CSH)-based materials achieve a compressive strength of 8.93 MPa by optimizing the liquid-to-solid ratio(0.3 mL/g) and incorporating 2% calcium sulfate dihydrate, making them suitable for non-weight-bearing bone defect repair (Lu et al., 2015). Key mechanical parameters to consider include compressive strength, fracture toughness, elastic modulus, and hardness (Thangavel and Elsen Selvam, 2022).

From a rheological perspective, ideal bioinks exhibit solid-like fluid behavior (Bercea, 2023). Rheological properties influence processing performance, post-implantation shape adaptability, structural formation, and cellular behavior. The printability and performance of bioinks can be evaluated based on gelation temperature, shear-thinning behavior, and viscoelastic propertiesv (O’Connell et al., 2020). Shear-thinning refers to the phenomenon where viscosity decreases as the shear rate increases, enabling the material to be injected through minimally invasive needles into bone defect sites. For example, at a liquid-solid ratio of 0.3 mL/g, the viscosity of α - hemihydrate calcium sulfate/sodium hyaluronate material significantly decreases, allowing for smooth injection and rapid recovery of viscosity to maintain shape stability (Lu et al., 2015). The viscoelastic modulus of materials must match that of natural bone tissue. Excessively high modulus inhibits cell migration, while insufficient modulus fails to provide mechanical support. Additionally, the initial and final setting times of materials must balance operational feasibility and defect-filling efficacy, ensuring neither injection blockage nor delayed pore formation.

The swelling effect is a “double-edged sword.” On one hand, swelling can expand the internal pores of scaffolds, enhance pore interconnectivity, and facilitate cell migration, nutrient diffusion, vascularization, improve mechanical compatibility, and regulate drug/growth factor release. On the other hand, excessive swelling may lead to risks such as pore collapse or structural deformation, imbalance in degradation rates, and compromised mechanical properties.

Biocompatibility directly governs the interaction between materials and host tissues, forming the biological foundation for bone regeneration. Surface chemistry and microtopography of materials significantly affect cellular behavior. Hydroxyapatite, resembling bone mineral composition, promotes osteoblast adhesion but often requires combination with COL-I or growth factors to enhance cellular activity (Song, 2008). Degradation products of synthetic materials may trigger inflammatory responses, necessitating surface modifications to mitigate toxicity. Certain bioactive materials, such as calcium sulfate, can also release calcium and phosphate ions to stimulate osteogenic differentiation.

In addition, the structure of the scaffold has an impact on performance. The right size and shape can be matched to the defect area, and it also optimises the mechanical distribution and the environment for cell growth. The structure of the scaffold can be optimised through bionic design and 3D printing of layered structures. Porosity and pore size affect cell migration, vascularisation and mechanical strength. 3D printing allows for customised design. Surface roughness affects cell adhesion, osteogenic differentiation and antimicrobial properties. Plasma treatment and chemical etching can regulate roughness. Mechanical fit, fatigue resistance and toughness affect long-term stability of scaffolds. Surface types such as hydrophilicity, surface chemistry and adhesion can influence cell behaviour and protein adsorption. They can be optimised by material composite, structural design, surface coating, and chemical modification.

In summary, numerous factors influence the efficacy of bone defect repair using biological scaffolds. When selecting scaffold materials and fabrication methods, it is critical to holistically evaluate these factors and tailor personalized scaffolds to meet specific clinical requirements.

## 5 Conclusion and perspective

The traditional surgical method is still the common method of bone defect repair, and autologous bone graft is considered as the gold standard of bone defect treatment. However, as people’s expectations for the prognosis of bone defects continue to increase, the effectiveness of traditional surgical methods is constantly being challenged. The emergence of bone tissue engineering technology provides a new idea for treatment. The combined application of 3D printing technology and synthetic composite biomaterial scaffolds is gradually maturing. The multipotent differentiation potential of MSCs also provides more possibilities for their participation as seed cells in bone defect repair. Various materials used to prepare biological scaffolds can directly or indirectly interact with cells involved in bone defect repair, such as MSCs and osteoblasts, to promote their adhesion, proliferation, osteogenic differentiation, and mineralization. Compared with single-material biological scaffolds, composite scaffolds have better mechanical strength and osteogenic properties. The combination of cytokines and biological scaffolds has unique advantages in antibacterial properties, promoting the differentiation of stem cells into bone and cartilage, facilitating bone tissue mineralization and neovascularization, and inhibiting adipogenesis. The combination of 3D printing technology and bone tissue engineering provides more options for the treatment of complex bone defects.

As a new approach, bone tissue engineering technology has certain advantages, but most of them are still in the theoretical and experimental stages, Further research and verification are needed to achieve clinical applications, and there are still many challenges that need to be solved.

The degradation rate of biodegradable scaffolds is difficult to control and the safety of biomaterials limit their wide application. Therefore, it is necessary to explore a scheme that can control the degradation rate of materials and improve materials.

Since the mechanical properties of biological scaffolds are different from those of normal human bones, the problems caused by insufficient mechanical strength or stress shielding after implantation need to be paid attention to. It can be considered to adjust the mechanical strength of scaffolds by changing the porosity and pore size of scaffolds and 3D printing personalized design.

Many scaffolds have problems of poor adhesion to cells and poor osteogenic ability. It is suggested to combine other materials or cytokines to improve the biocompatibility and osteogenic performance of composite scaffolds.

Further research is needed on how to differentiate MSCs with multi-directional differentiation potential towards the direction beneficial for bone defect repair, as well as how to reasonably accelerate the mineralization of osteoblasts and the formation of neovascularization by ECs.

The currently investigated single-material bioscaffolds have limitations and often do not meet the requirements for an ideal bioscaffold. Inadequate mechanical properties, mismatch between degradation rate and bone regeneration, immunogenicity risk, long-term safety issues and difficulty in clinical translation all limit the application of bioscaffolds. For example, natural polymer scaffold systems have advantages in terms of biocompatibility and degradability, but their mechanical properties, degradation match, immunogenic risk and processing complexity remain major bottlenecks.

Blood circulation disorder may lead to hypoxia and nutritional deficiency of transplanted cells, and then affect the repair effect. In the process of bone tissue engineering repair, how to ensure unimpeded blood circulation between the new tissue and the surrounding tissue is a key problem.

How to safely and effectively transplant the new tissue constructed *in vitro* into the body, and make it form a good combination and interaction with the surrounding tissues is another difficult problem faced by bone tissue engineering technology. At present, there is still a lack of unified standards and norms about the specific manners, timing and methods of *in vivo* transplantation of newborn tissue.

3D printed scaffolds are mostly still in the animal model validation stage, with less data from early clinical trials. Challenges are encountered in clinical translation. There are many factors affecting clinical translation, such as large batch-to-batch variation in materials, lack of established uniform evaluation criteria and lack of standardization and stability. The potential toxicity of degradation products needs to be systematically evaluated, and long-term safety needs to be verified. High cost of high-precision technical equipment, such as DLP, makes it difficult to meet large-scale clinical needs. There are less materials that have been approved for clinical application, including titanium, HA, β-TCP, PLA and other materials. Table 6 lists some of the bone tissue engineering scaffold systems that have been approved for clinical application.

**TABLE 6:** Clinically approved scaffold systems for bone tissue engineering application.

Product Name	Approval Year/Region	Material Composition	Indications
INFUSE^®^ Bone Graft	2002 (FDA, United States)	Recombinant human BMP-2^+^ collagen sponge scaffold	Spinal fusion, open tibial fractures
Collagraft^®^	1990s (FDA, United States)	Collagen + HA/TCP composite	Bone defect filling, fracture repair
NovaBone^®^	2000s (FDA, United States)	Bioactive glass (SiO_2_-CaO-P_2_O_5_)	Alveolar bone defects, maxillofacial bone repair
Orthoss^®^	2000s (CE, EU)	Bovine-derived bone mineral (HA)	Bone defect repair, spinal fusion
chronOS^®^	2006 (CE, EU)	β-TCP	Bone cyst filling, traumatic bone defects
Osteoplug^®^	2010s (NMPA, China)	PLA composite	Bone nails/plates for minimally invasive orthopedic surgery
3D ACT Artificial Bone	2024 (NMPA, China)	Customized titanium alloy scaffold	Complex bone defect repair
Vitoss^®^	2000s (FDA, United States)	β-TCP	Bone defect filling, spinal fusion
Inductigraft^®^	2010s (FDA, United States)	Collagen + calcium phosphate composite	Bone defect filling, spinal fusion

Although 3D-printed bone tissue engineering scaffolds still face significant challenges in materials, technology, and clinical translation, their immense potential in personalized medicine, regenerative medicine, and smart materials cannot be overlooked. A comprehensive analysis of their developmental trajectory will facilitate technological breakthroughs, clinical implementation, and clearer future directions.

① New materials are continuously developed and their properties are gradually optimized. Multifunctional composites are emerging, and active elements such as strontium and magnesium are doped to enhance the bioactivity and osteoinductive ability of scaffolds. The application of two-dimensional materials is also gradually increasing. For example, black phosphorus has been used to regulate the immune microenvironment due to the promotion of bone mineralization by it's degradation product PO_4_^3-^.
② Through composite material design, functional filler incorporation, chemical modification, and coating techniques for material and surface modifications of scaffolds, we can enhance their mechanical strength, degradation behavior, bioactivity, and cytocompatibility, thereby better meeting the requirements for bone defect repair.
③ There are also more ideas for the design of scaffold structure. The triply periodic minimal surface(TPMS) structure can disperse the stress and improve the mechanical properties of the scaffold. Combining 3D printing and microfluidic technology, the construction of through holes can promote angiogenesis and cell migration. Some scaffolds modified by surface functionalization have an important impact in regulating the immune microenvironment at bone defects.
④ The degradation rate of scaffolds is a key link between materials science and bone regenerative medicine. The dynamic balance between it and the rate of bone regeneration directly determines the repair effect. Most of the existing materials undergo linear degradation, making it difficult to simulate the nonlinear rate requirements for bone healing. Personalized degradation plans are required due to differences in patient age, defect location, and overall metabolic status. Through material characterization modification, combined with dynamic response technology and personalized manufacturing, it is expected to achieve precise synchronization of “degradation regeneration” and promote the transition of bone defect repair from structural reconstruction to functional regeneration.
⑤ In terms of 3D printing technology, the demand for high-precision and multi-material synergistic printing has increased significantly. Digital light processing is more advantageous in realizing precise molding of TPMS structures, which is suitable for the repair of complex bone defects. Mechanics-assisted printing strategy can dynamically regulate cell loading through compressive strain, balancing the printing precision and cell survival rate.
⑥ Intelligent and dynamic modulation technologies are also evolving. 4D printing technology has obvious advantages in developing scaffolds responsive to temperature, pH or enzyme environments, realizing *in vivo* adaptive morphology adjustments and controlled release of drugs. Combining macroscopic scaffold printing with nanoscale surface modification can enhance cell adhesion and signaling.
⑦ Meanwhile, we should also pay attention to the need for deep integration of biomaterials and cells. The deep integration of biomaterials and cells can be realized by 3D printing, electrostatic spinning and microfluidic technology. Mechanical microenvironment regulation and the control of growth factor release facilitate the differentiation of loaded cells in the scaffold. In the future, we need to break through the technical bottleneck of material-cell interaction mechanism, dynamic functional design and clinical scale production. Promote the wide application of personalized bone regeneration solutions.

Regarding future breakthrough directions, experimental validation should focus on the feasibility of integrating smart materials with precision medicine. Develop AI-driven scaffold design platforms that customize porosity and mechanical parameters based on patient CT data. Combine gene-editing technologies to engineer stem cell-scaffold complexes carrying osteogenic genes. Multidisciplinary cross-innovation is also essential. Integrate computational fluid dynamics and molecular dynamics to simulate and predict the long-term rheological behavior and regenerative effects of scaffolds. Exploring conductive biomaterials and their application in regulating cellular metabolism and osseointegration through electrical stimulation presents a promising research Frontier.
